# Innate immunity, cytokine storm, and inflammatory cell death in COVID-19

**DOI:** 10.1186/s12967-022-03767-z

**Published:** 2022-11-22

**Authors:** Rajendra Karki, Thirumala-Devi Kanneganti

**Affiliations:** Department of Immunology, St. Jude Children’s Research Hospital, MS #351, 262 Danny Thomas Place, Memphis, TN 38105-3678 USA

**Keywords:** IFN, TNF, Pyroptosis, Necroptosis, PANoptosis, PANoptosome

## Abstract

The innate immune system serves as the first line of defense against invading pathogens; however, dysregulated innate immune responses can induce aberrant inflammation that is detrimental to the host. Therefore, careful innate immune regulation is critical during infections. The coronavirus disease 2019 (COVID-19) pandemic is caused by severe acute respiratory syndrome coronavirus 2 (SARS-CoV-2) and has resulted in global morbidity and mortality as well as socio-economic stresses. Innate immune sensing of SARS-CoV-2 by multiple host cell pattern recognition receptors leads to the production of various pro-inflammatory cytokines and the induction of inflammatory cell death. These processes can contribute to cytokine storm, tissue damage, and acute respiratory distress syndrome. Here, we discuss the sensing of SARS-CoV-2 to induce innate immune activation and the contribution of this innate immune signaling in the development and severity of COVID-19. In addition, we provide a conceptual framework for innate immunity driving cytokine storm and organ damage in patients with severe COVID-19. A better understanding of the molecular mechanisms regulated by innate immunity is needed for the development of targeted modalities that can improve patient outcomes by mitigating severe disease.

## Introduction

In December 2019, a cluster of pneumonia cases with unknown causes was first reported in Wuhan, China. The first complete gene sequence from patient samples was obtained in January 2020 and showed a novel beta coronavirus as the causative agent for the cases [1]. While initially reported as 2019-nCoV, the virus has now been classified as severe acute respiratory syndrome coronavirus (SARS-CoV)-2, and the resulting disease is named coronavirus disease 2019 (COVID-19). In March 2020, the World Health Organization (WHO) declared the COVID-19 outbreak a global pandemic [2]. As of October 2022, COVID-19 had caused over 6.5 million deaths from among 620 million confirmed cases [3]. COVID-19 is associated with a myriad of clinical manifestations. Some patients remain asymptomatic, but most experience mild symptoms associated with respiratory tract infections such as fever, headache, and cough. However, patients with severe COVID-19 can have systemic inflammation and tissue damage as well as acute respiratory distress syndrome (ARDS), thromboembolism, neurological manifestations, cardiac injury, cytokine storm, and organ failure, which can be lethal (Table 1) [4, 5]. The likelihood of developing severe COVID-19 depends on viral factors, such as the specific variant of the virus, as well as patient-specific factors like age, genetic polymorphisms, ethnicity, vaccination status, and comorbidities (e.g., diabetes, hypertension, obesity, and other conditions) [6].

**Table 1:** Clinical manifestations of COVID-19: Several tissues, organs, and body systems are affected in patients with COVID-19

Lungs	Heart	Liver	Skin	Kidney
Pneumonitis	Hypotension	Hepatomegaly	Urticaria	Hematuria
Pulmonary edema	Arrythmias	Increased AST, ALT, LDH	Rash	Proteinuria
Dyspnea	Cardiomyopathy	Elevated bilirubin	Edema	Acute kidney injury
Hypoxemia	Ischemia	Liver failure	Vesicles	Kidney failure
ARDS	Cardiogenic shock			

Nervous system	Gastro-intestinal system	Vascular lymphatic system	Rheumatologic system	Constitutional symptoms
Confusion	Nausea	Anemia, cytopenia	Vasculitis	Fever
Delirium	Vomiting	Coagulopathy	Arthritis	Headache
Ataxia	Diarrhea	Hyperferritinemia	Arthralgia	Fatigue
Seizures	Abdominal pain	Increased CRP		Anorexia
Anosmia	Hemorrhage	Hemorrhage		
Stroke		Endothelial damage		

*ALT* alanine aminotransferase; *ARDS*; acute respiratory distress syndrome; *AST* aspartate aminotransferase; *CRP* C-reactive protein; *LDH* lactate dehydrogenase

Innate immunity is the first line of defense against any invading pathogen, including SARS-CoV-2. A broad range of innate immune cells, including macrophages, neutrophils, dendritic cells, natural killer (NK) cells, eosinophils, basophils, and innate lymphoid cells (ILCs), are activated during SARS-CoV-2 infection and COVID-19 pathogenesis. Innate immune sensing of SARS-CoV-2 is mediated by recognition of the viral pathogen-associated molecular patterns (PAMPs) by multiple pattern recognition receptors (PRRs) leading to the production of cytokines, including interferons (IFNs), and chemokines. Host PRRs include membrane receptors, such as the Toll-like receptors (TLRs) and C-type lectin receptors (CLRs), as well as cytosolic receptors, such as the NOD-like receptors (NLRs), absent in melanoma 2 (AIM2)-like receptors (ALRs), and retinoic acid-inducible gene-I (RIG-I)-like receptors (RLRs) [7]. Emerging studies have shown that during SARS-CoV-2 infection, these receptors induce innate immune-mediated cytokine production and cell death. For instance, SARS-CoV-2 directly stimulates infected epithelial cells and circulating myeloid cells to release several pro-inflammatory cytokines [8, 9]. Although early inflammatory responses are crucial to limit viral replication [10], coronaviruses have evolved strategies to escape innate immune sensing by avoiding PRR activation and by interfering with downstream IFN responses [11]. In contrast, critical cases of COVID-19 are characterized by an excessive inflammatory response both in the lungs and bloodstream [4, 5]. In these cases, the combination of cytokines, particularly tumor necrosis factor (TNF) and interferon gamma (IFN-γ), triggers inflammatory cell death called PANoptosis and consequently results in cytokine storm [5, 12]. Overall, while early innate immune-mediated inflammatory responses are critical for host defense against viral infection, late inflammatory responses, if not controlled, can lead to tissue damage and organ failure [13]. Therefore, strategies to modulate innate immune activation have strong therapeutic potential in COVID-19. Several agents targeting innate immunity have been repurposed for the treatment of COVID-19, but there have been mixed responses to these therapeutics to date [14].

In this review, we discuss the contribution of multiple PRRs in detecting SARS-CoV-2 to activate the innate immune response and produce inflammatory cytokines. We also highlight how innate immune sensing and activation can lead to pathology, including how TNF and IFN-γ–mediated PANoptosis can drive cytokine storm and severity in COVID-19. Moreover, we discuss immunomodulatory therapeutic strategies in COVID-19 and how the timing of IFN therapy influences patient outcomes. Continuing to improve our understanding of the innate immune system and inflammatory cell death will be critical to allow translation from molecular mechanisms to therapeutic strategies for this pandemic as well as future outbreaks.

## Innate immune sensing and signaling in response to SARS-CoV-2

SARS-CoV-2 infection is sensed by a variety of host PRRs. The recognition of PAMPs or damage-associated molecular patterns (DAMPs) by these PRRs induces multiple inflammatory signaling pathways, including the upregulation of innate immune genes, the induction of innate immune-mediated cell death, and the production of inflammatory cytokines and chemokines. While these responses can be helpful in clearing the virus or infected cells, activation of PRRs can also lead to pathogenic inflammation and tissue damage.

### TLR sensing and signaling in response to SARS-CoV-2

To date, 10 TLR family members, designated as TLR1–10, have been identified in humans. TLR expression can be detected in the respiratory tract, though the level of expression varies depending on the cell population. For example, TLR4 is expressed abundantly in macrophages, and TLR3 is more abundant in NK cells [15]. Downstream of TLR engagement by PAMPs and DAMPs, TLRs mediate their signaling through adaptor molecules. TLR3 exclusively signals via the adaptor protein Toll/interleukin-1 (IL-1) receptor (TIR) domain-containing adapter-inducing IFN-β (TRIF), while TLR4 signals through either myeloid differentiation primary response 88 (MyD88) or TRIF, and the remaining TLRs use MyD88 [16]. MyD88 signaling activates nuclear factor-κB (NF-κB), mitogen-activated protein kinase (MAPK), and IFN regulatory factor (IRF) signaling cascades for transcriptional upregulation of several pro-inflammatory cytokines, IFNs, and innate immune sensor genes, such as NOD-like receptor protein 3 (NLRP3). TRIF signaling is primarily involved in the production of IFNs to induce antiviral functions [16].

**Fig. 1:**
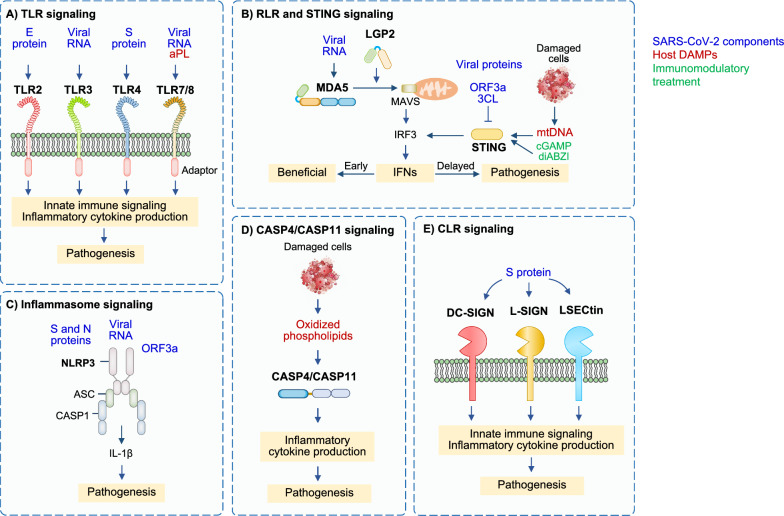
Pattern recognition receptor signaling and potential innate immune-mediated pathogenesis during SARS-CoV-2 infection. **A** Toll-like receptor (TLR) signaling: Different TLRs recognize diverse SARS-CoV-2 components. TLR2 and TLR4 recognize E protein and S protein, respectively; TLR3, TLR7, and TLR8 sense viral RNA. TLR7 and TLR8 also recognize antiphospholipid antibodies (aPL). Sensing of SARS-CoV-2 components leads to activation of innate immune signaling and production of pro-inflammatory cytokines, which can eliminate virus but also drive COVID-19 severity. **B** Retinoic acid-inducible gene-I (RIG-I)-like receptor (RLR) and stimulator of IFN genes (STING) signaling: Melanoma differentiation-associated protein 5 (MDA5) senses viral RNA. STING can be activated by STING agonists or mitochondrial DNA (mtDNA) released by damaged cells. Signaling through MDA5 and STING engages interferon (IFN) regulatory factor 3 (IRF3) activation for the production of IFNs. Early on, IFNs are important to clear viruses. Delayed production of IFNs is pathogenic. **C** Inflammasome signaling: The nucleotide-binding oligomerization domain-like receptor protein 3 (NLRP3) inflammasome is assembled following sensing of spike (S) and nucleocapsid (N) proteins, viral RNA, and open reading frame 3a (ORF3a). This assembly leads to the production of interleukin (IL)-1β, which has been reported to drive COVID-19 pathology. **D** Caspase-4 (CASP4)/caspase-11 (CASP11) signaling: CASP4 and the murine homolog CASP11 sense oxidized phospholipids released from damaged cells and produce pro-inflammatory cytokines, which can drive COVID-19 pathology. **E** C-type lectin receptor (CLR) signaling: S protein from SARS-CoV-2 is sensed by CLRs such as dendritic cell specific intercellular adhesion molecule-3-grabbing non-integrin (DC-SIGN), liver/lymph node-specific intercellular adhesion molecule-3-grabbing non-integrin (L-SIGN), and liver sinusoidal endothelial cell lectin (LSECtin) to induce innate immune signaling and the production of pro-inflammatory cytokines, which can be pathogenic in COVID-19

In the context of SARS-CoV-2 infection, multiple TLRs can sense diverse components of the infection, both in the form of viral PAMPs and host DAMPS, to induce innate immune activation (Fig. 1A). TLR1, TLR2, and TLR6 bind viral proteins, and TLR2 can form heterodimers with TLR1 and TLR6. Polymorphism in *TLR1* is associated with the development of ARDS in sepsis [17], though its role in the development of COVID-19 requires further study. TLR2 senses the SARS-CoV-2 envelope (E) protein, and expression of *TLR2* and its adaptor *MyD88* are associated with COVID-19 disease severity [18]. TLR2-deficient murine macrophages or TLR2 inhibitor-treated human macrophages show reduced activation of pro-inflammatory signaling pathways and reduced production of cytokines in response to SARS-CoV-2 E protein [18]. The level of IL-6 in serum is decreased in *Tlr2*^–/–^ mice compared with wildtype mice when treated with the E protein [18], suggesting inflammatory responses triggered by the viral E protein depend on TLR2 signaling. Moreover, TLR2 inhibition protects against SARS-CoV-2–induced lethality in K18-human angiotensin-converting enzyme 2 (K18-hACE2) transgenic mice [18], indicating that TLR2 contributes to disease progression in COVID-19. Further, a single-cell computational analysis identified *TLR2* as a key gene involved in exacerbating the hyperinflammatory response seen in patients with severe COVID-19 [19]. Additionally, decreased IL-6 production was found in E protein-stimulated human peripheral blood plasmacytoid dendritic cells (pDCs) where *TLR2* had been deleted when compared to pDCs with intact *TLR2* [20]. Further studies are required to establish the E protein as a ligand for direct binding to TLR2. In contrast to the pathogenic role of TLR2 in inducing excess inflammation, intranasal administration of INNA-051, a TLR2/TLR6 agonist, reduces the level of viral RNA in nose and throat swabs from SARS-CoV-2–infected ferrets [21], suggesting that stimulation of TLR2 in specific tissues may be beneficial in preventing the development of COVID-19 by reducing SARS-CoV-2 transmission. However, the administration of INNA-051 fails to induce inflammatory responses in the ferrets, indicating that INNA-051 may exert antiviral effects independent of TLR2 activation [21].

Roles for other TLRs in sensing SARS-CoV-2 have also been postulated based on their critical roles in other coronavirus infections. In the context of SARS-CoV, for example, TLR3 signaling has a protective role [22–25]. Mice deficient in TLR3 show increased viral burden and impaired pulmonary function upon infection with mouse-adapted SARS-CoV (MA15) [25]. Consistently, TLR3 activation by poly I:C, a synthetic dsRNA used to mimic viral nucleic acids and model innate immune responses, improves the survival of aged mice and reduces pathological changes and viral loads in the lungs following infection with MA15 [23]. Moreover, inhibition of TLR3 leads to decreased production of inflammatory cytokines and IFNs in SARS-CoV-2–infected Calu-3/MRC-5 multicellular spheroids [26]. Similarly, administration of poly I:C improves survival in K18-hACE2 transgenic mice during SARS-CoV-2 infection by reducing viral load and inflammation in the lungs and brains [27]. These findings suggest there may be a protective effect of TLR3 signaling during COVID-19. Additionally, reduced expression of *TLR3* in peripheral blood has been associated with worse outcomes in patients with severe COVID-19 [28]. This is further supported by a study which analyzed patients with severe COVID-19 and showed a positive correlation between the severity of disease and *TLR3* inborn errors [29]. However, a follow-up study could not reproduce these results [30]. In addition, a *TLR3* (rs3775290) polymorphism has been associated with an increased risk of pneumonia in patients with COVID-19 [31]. Further studies are needed to define the role of TLR3 in SARS-CoV-2 infection and the development of COVID-19.

Potential roles for TLR4 and TLR7/8 have also been suggested. In an in-silico study, TLR4 had the highest affinity for S1 of the SARS-CoV-2 spike (S) protein [32], which may be due to the interaction of TLR4 with oligomannose and glycan structures found on the surface of the S protein. Additionally, *TLR4* polymorphisms are associated with COVID-19 severity; the *TLR4* minor alleles 299Gly (G) and 399Ile (T) are associated with increased likelihood of severe COVID-19 and the risk of developing cytokine storm [33]. In murine models, *Tlr4*^–/–^ macrophages from mice had reduced *Il1b* gene expression compared to that of wildtype cells in response to S protein [34]. However, because S protein has an affinity for lipopolysaccharide (LPS) [35], it is possible that LPS contamination from the system used to express and purify the recombinant S protein in these studies contributed to the TLR4-dependent gene expression. Therefore, whether TLR4 can directly sense S protein or not requires further confirmation. In addition, TLR7/8 may also be involved in SARS-CoV-2 infection. TLR7/8 have long been studied for their role in antitumoral immune responses due to their ability to provoke rapid production of IFNs and cytokines [36]. A bioinformatic analysis reported that a large number of single stranded RNA (ssRNA) fragments are present in SARS-CoV-2, even more so than in SARS-CoV [37]. These ssRNA fragments can be recognized by endosomal TLR7/8 to generate antiviral immunity [38]. Furthermore, functional *TLR7* genetic variants have been linked to COVID-19 severity in multiple studies [31, 39, 40]. Specifically, a *TLR7* (rs179008) polymorphism has been associated with increased risk of pneumonia in patients with COVID-19 [31], and deleterious *TLR7* variants are associated with more severe disease [39, 40]. Given that *TLR7* is found on the X-chromosome, TLR7 may influence gender-based differences in COVID-19 susceptibility and severity. Males are nearly two times as likely to develop respiratory failure or die from COVID-19 as females are [41, 42]. TLR7/8 may also recognize antiphospholipid antibodies (aPL) [43, 44], which have been shown to be upregulated in patients with severe and critical COVID-19 [45, 46]. Furthermore, TLR7-deficient human peripheral blood pDCs show decreased IFN production compared with TLR7-sufficient pDCs following SARS-CoV-2 infection [20]. Additional investigations are needed to verify the roles of these and other TLRs in response to SARS-CoV-2 infection.

### RLR sensing and signaling in response to SARS-CoV-2

In addition to sensing by TLRs, viral RNA can be detected by RLRs such as melanoma differentiation-associated protein 5 (MDA5), retinoic acid-inducible gene (RIG)-I, and laboratory of genetics and physiology (LGP)-2 (Fig. 1B) [47, 48]. Sensing through RLRs leads to their interaction with the adaptor protein mitochondrial antiviral signaling (MAVS) to form a complex that activates IFN production via TNF receptor-associated factor 3 (TRAF3), TRAF-associated NF-κΒ (TANK)-binding kinase 1 (TBK1), IκB kinase (IKK), and IRF3 signaling [49, 50]. In addition, one study showed that RIG-I is required for the induction of NF-κB-sensitive genes, interleukin (IL)-6, and TNF, while MDA5 is not in Calu-3 cells during SARS-CoV-2 infection [51]. The earlier, more pronounced antiviral response to SARS-CoV-2 infection seen in children compared to adults has been suggested to be associated with higher basal expression of MDA5 and RIG-I in the epithelial cells of the upper airway as well as macrophages and dendritic cells in children [52]. This could be a possible explanation for why children have lower infection rates for SARS-CoV-2 and a lower risk for disease progression compared with adults. Screening of putative sensors involved in the sensing of RNA virus infection has found that MDA5 and LGP2 primarily regulate IFN induction in lung epithelial cells during SARS-CoV-2 infection [47]. Silencing *MDA5*, *LGP2*, and *MAVS* in lung epithelial cells reduces the expression of IFN-β during SARS-CoV-2 infection in vitro [47, 48]. Similarly, gene silencing of *MDA5* in Calu-3 cells decreases IFN-β production compared to controls during infection with SARS-CoV-2 [51]. Abrogating SARS-CoV-2 sensing via MDA5 and MAVS depletion also reduces cell death, suggesting that cell death is mediated by the host response rather than by direct virus-induced damage [51]. In contrast, the role of RIG-I in sensing SARS-CoV-2 is currently inconclusive. One study found that silencing *RIGI* in Calu-3 cells abolishes the production of IFN-β and other pro-inflammatory cytokines during infection with SARS-CoV-2 [51]. In addition, SARS-CoV-2–infected RIG-I–deficient HEK-293 cells fail to induce IFN-β mRNA expression (53). In contrast, another study showed that silencing *RIGI* in Calu-3 cells does not inhibit the induction of IFN-β in response to SARS-CoV-2 infection [47]. RIG-I utilizes its helicase domain to recognize the 3’ untranslated region of SARS-CoV-2 genomic RNA, which differs from RIG-I’s canonical C-terminal domain sensing of viral RNA. This new mode of RIG-I recognition fails to activate its ATPase; it also does not activate the classical MAVS signaling pathway. These signaling differences could potentially explain the dispensable role of RIG-I in IFN-β production during SARS-CoV-2 infection [54]. Additional studies will be required to fully understand the role of RIG-I in response to SARS-CoV-2. However, it is known that SARS-CoV-2 encodes inhibitors of both RIG-I and MDA5, suggesting that RIG-I and MDA5 likely possess some cell type-specific functions. A better understanding of the roles of RIG-I and MDA5 in different cell types will aid in the development of more efficient COVID-19 therapeutics.

### NLR and inflammasome sensing and signaling in response to SARS-CoV-2

Several NLRs have been implicated in the production of IFNs and inflammatory cytokines during SARS-CoV-2 infection. For instance, silencing of an intracellular sensor of bacterial peptidoglycans, nucleotide-binding oligomerization domain-containing protein 1 (NOD1; also known as NLRC1), in Calu-3 cells during SARS-CoV-2 infection reduces the expression of IFN-β [47]. NLRC1 may mediate SARS-CoV-2 infection-induced NF-κΒ activation or directly bind viral RNA and regulate the MDA5-MAVS complex formation to modulate IFN-β production [55]. Several reports have demonstrated that NLRP3 is also involved in coronavirus infections (Fig. 1C). NLRP3 is a canonical inflammasome sensor, and it forms a multiprotein complex with the adaptor apoptosis-associated speck-like protein containing a caspase activation and recruitment domain (ASC) and caspase-1 in response to PAMP/DAMP sensing. Inflammasome formation results in the activation of caspase-1 to cleave its substrates, including the pro-inflammatory cytokines IL-1β and IL-18 to produce their bioactive forms. Caspase-1 also cleaves gasdermin D (GSDMD) to release its N terminal fragment to form pores in the membrane and induce cell death, pyroptosis [56]. The SARS-CoV-2 N protein is thought to bind to GSDMD to inhibit pyroptosis [57]. However, other studies have suggested that NLRP3 inflammasome activation and pyroptosis still occur during SARS-CoV-2 infections. NLRP3 deficiency inhibits caspase-1 and GSDMD activation in a murine coronavirus infection model with mouse hepatitis virus (MHV) [58], indicating that coronaviruses induce NLRP3 inflammasome assembly. Additionally, microscopy of monocytes and lung tissue samples from patients with COVID-19 show the formation of NLRP3 and ASC puncta, suggesting the formation of NLRP3 inflammasomes in these patients [59]. Moreover, SARS-CoV-2–infected human primary monocytes show NLRP3-dependent caspase-1 cleavage, GSDMD cleavage, and IL-1β maturation [59, 60]. NLRP3 is upregulated and activated by multiple SARS-CoV-2 PAMPs, including GU-rich RNAs, E, N, and open reading frame (ORF) 3a proteins [18, 61–63]. In response to the SARS-CoV-2 S protein, NLRP3 expression and IL-1β release are upregulated in macrophages from patients with COVID-19 but not in macrophages from healthy patients [64]. Additionally, SARS-CoV-2 N protein interacts directly with NLRP3, thereby facilitating the binding of NLRP3 with ASC [62]. N protein-mediated lung injury, inflammation, and death in mice is reduced upon treatment with an NLRP3 inflammasome inhibitor, MCC950, or a caspase-1 inhibitor, Ac-YVAD-cmk [62]. MCC950 treatment also alleviates excessive lung inflammation and COVID-19–like pathology in adeno-associated virus (AAV)-hACE2 transgenic mice, indicating that the NLRP3 inflammasome induces excessive inflammatory responses during SARS-CoV-2 infection [65]. This detrimental effect of NLRP3 during SARS-CoV-2 infection is further supported by the finding that the severe pathology induced by SARS-CoV-2 in lung tissues is reduced in *Nlrp3*^–/–^ mice compared to wildtype mice [65]. Activation of the NLRP3 inflammasome is also seen in peripheral blood mononuclear cells (PBMCs) and tissues of postmortem patients with COVID-19 upon autopsy [59]. Moreover, higher levels of inflammasome-dependent products, such as IL-18 and active caspase-1, are associated with disease severity and poor clinical outcome [59]. Collectively, these data suggest that SARS-CoV-2 activates the NLRP3 inflammasome. Additionally, during SARS-CoV-2 infection, the expression of proteins involved in non-canonical NLRP3 inflammasome signaling, through caspase-11 and caspase-4, are upregulated in the lungs of mice and humans, respectively. Deficiency of caspase-11, but not GSDMD, an executioner of pyroptosis, reduces disease severity in SARS-CoV-2–infected mice [66], suggesting that caspase-11 (or caspase-4/5 in humans) may promote disease severity in COVID-19 independently of pyroptosis. Caspase-4/11 can be activated by oxidized phospholipids that are produced in damaged tissues (Fig. 1D) [67]. Thus, it is possible that this activation occurs during SARS-CoV-2 infection. Oxidized phospholipids are reportedly upregulated in patients with COVID-19 [68, 69], and outside the context of COVID-19, they induce cytokine release in a caspase-4/11–dependent manner without requiring GSDMD-dependent cell death [67].

Beyond NLRP3, other inflammasomes have also been implicated in SARS-CoV-2 infection. Colocalization of the AIM2 inflammasome with ASC specks in monocytes from patients with COVID-19 has been visualized using confocal microscopy [70]. AIM2 activation by SARS-CoV-2 is unexpected, although there is a report suggesting AIM2 activation by RNA viruses occurs in rare cases through an unclear mechanism [71]. AIM2 might sense host mitochondrial DNA, as mitochondrial membranes are damaged during cell death [72].

### cGAS and STING sensing and signaling in response to SARS-CoV-2

The presence of cytosolic DNA during infection activates the cyclic GMP-AMP synthase (cGAS) and stimulator of IFN genes (STING) signaling pathway, which is critical for limiting the replication of viruses (Fig. 1B) [73–76]. Sensing of cytosolic DNA through cGAS generates the production of cyclic GMP-AMP, which acts as a second messenger to bind and activate STING [76]. Once activated, STING signals through TBK1 and IRF3 to regulate the transcription of innate immune genes, including cytokines and IFNs [73, 75, 76]. Analysis of the skin lesions of patients with COVID-19 shows a STING-dependent type I IFN signature that is primarily mediated by macrophages adjacent to areas of endothelial cell damage [77]. Moreover, cGAS-STING activity has been detected in lung samples from patients with severe COVID-19. The finding that STING regulates IFN production and endothelial cell death in response to mitochondrial DNA release correlates well with the studies of endotheliopathy and vascular damage due to gain-of-function mutations in STING [78] or after administration of highly potent STING agonists [79]. This possibly explains the presence of vascular damage and coagulopathy in patients with severe COVID-19. Moreover, another study has shown that the cGAS-STING pathway contributes to the production of NF-κB–dependent pro-inflammatory cytokines in SARS-CoV-2–infected epithelial cells [80]; however, cGAS-STING is not involved in IFN production in these cells [80]. This non-canonical function of the cGAS-STING pathway in epithelial cells may dysregulate the pro-inflammatory response.

Treatment of K18-hACE2 transgenic mice with a selective STING inhibitor before and throughout the course of SARS-CoV-2 infection or as a therapeutic agent given after the initiation of infection reduces inflammation and improves survival [77]. In contrast, STING agonists such as cyclic guanosine monophosphate-adenosine monophosphate (cGAMP) or diamidobenzimidazole (diABZI) have also been shown to limit SARS-CoV-2 infection in mouse models when given prophylactically or early in infection [81–83]. Endotracheal administration of STING agonists in mice triggers a lung inflammatory response and cell death [84]. Together, these data suggest that early STING activation to induce IFN production may be beneficial, while later in infection, pro-inflammatory cytokines produced by epithelial cells, endothelial cells, and macrophages via the cGAS-STING pathway provoke pathological effects.

### CLR sensing and signaling in response to SARS-CoV-2

CLRs tailor their immune responses to pathogens by carbohydrate-based PAMP sensing (Fig. 1E) [85]. CLRs can have diverse functions in innate immune activation, ranging from the modulation of other PRR signaling pathways and phagocytosis for antigen presentation to the induction of inflammatory cytokine production [86]. An ectopic expression screen focused on myeloid cell receptors showed that several CLRs such as dendritic cell specific intercellular adhesion molecule-3-grabbing non-integrin (DC-SIGN), liver/lymph node-specific intercellular adhesion molecule-3-grabbing non-integrin (L-SIGN), liver sinusoidal endothelial cell lectin (LSECtin), asialoglycoprotein receptor (ASGR1), and c-type lectin domain family 10 member A (CLEC10A) are glycan-dependent binding partners of the SARS-CoV-2 S protein [87]. These receptors do not support active viral replication, but they can play a role in disease severity by contributing to robust inflammatory responses in myeloid cells [87]. Additionally, multiple studies show that LSECtin and DC-SIGN can serve as receptors for the S glycoprotein of SARS-CoV-2 [88, 89]. Anti-S nanobody treatment not only blocks ACE2-mediated infection but also inhibits myeloid receptor-mediated proinflammatory responses; this is likely because the S protein is recognized by both CLRs and ACE2 [87]. In addition, PM26, a glycomimetic antagonist of DC-SIGN, inhibits the interaction of the S protein with the lectin receptor and blocks DC-SIGN–mediated SARS-CoV-2 trans-infection of Vero E6 cells [88]. In addition to their roles in sensing to activate innate immune signaling, CLRs, especially DC-SIGN and L-SIGN, assist in the entry of viruses such as human immunodeficiency virus (HIV), cytomegalovirus, dengue, Ebola, and Zika virus into host cells [90–93]. SARS-CoV-2 S protein binds with CLRs in a calcium-dependent manner to facilitate the internalization of the virus [94]. Single cell RNA sequencing analyses indicate that CLRs are highly expressed in the innate immune cells of patients with severe COVID-19 [87, 94], and a proteomic profiling study indicates that DC-SIGN is a mediator of genetic risk in COVID-19 [95]. Overall, the biological relevance of the interaction of CLRs with SARS-CoV-2, especially in the context of innate immune activation, remains understudied.

## Cell death in COVID-19 pathogenesis

One of the key outcomes of innate immune sensing is cell death to clear infected or damaged cells. SARS-CoV-2 infection and the resulting cytokine production sensitize multiple cell types, including epithelial, endothelial, and immune cells, to cell death through diverse mechanisms [96]. SARS-CoV-2 infection can induce cell–cell fusion and syncytia formation in the lungs and other tissues of infected patients [97–99], as well as in in vitro culture systems [100–102]. The multinucleate syncytia formed by SARS-CoV-2 infection can internalize lymphocytes to form typical cell-in-cell structures, leading to the death of internalized cells [103]. Additionally, the different components of SARS-CoV-2 have been reported to induce various forms of cell death. The SARS-CoV-2 S protein induces syncytia formation in ACE2-expressing cells, and the resulting cell death has been characterized as GSDME-dependent pyroptosis [102]. Infected pneumocytes also undergo pyroptosis to release multiple DAMPs and cytokines [104]. In addition, cells expressing SARS-CoV-2 ORF3a show more cleavage of caspase-9 and caspase-3 than control cells, suggesting that SARS-CoV-2 ORF3a induces apoptosis [105]. Moreover, several reports have indicated SARS-CoV-2–mediated activation of necroptosis in multiple cell types, such as macrophages and epithelial cells [13, 106].

Due to the presence of multiple PAMPs in SARS-CoV-2 that can be sensed by the cell to activate signaling, multiple cell death molecules can be involved in creating redundancy in the induction of cell death. Indeed, analysis of postmortem lung sections of deceased patients with COVID-19 identified the presence of molecules associated with pyroptosis, apoptosis, and necroptosis [106, 107]. These findings suggest that SARS-CoV-2 infection induces PANoptosis, a unique innate immune inflammatory cell death pathway regulated by PANoptosomes, complexes that integrate molecules from other cell death pathways. The totality of biological effects in PANoptosis cannot be individually accounted for by pyroptosis, apoptosis, or necroptosis alone [12, 13, 108–114, 115, 116]. To date, two prototypical PANoptosomes have been biochemically identified: the Z-DNA binding domain protein 1 (ZBP1)-PANoptosome [113, 117], and the AIM2-PANoptosome [118]. These PANoptosomes have different sensors but share many core cell death proteins which have been implicated in SARS-CoV-2 infection and COVID-19 [106, 107]. These molecular connections will be discussed in depth in subsequent sections.

## IFN signaling in COVID-19 pathogenesis

In addition to cell death, IFN production is another key outcome of innate immune activation and is an integral component of antiviral responses. There are three IFN families: type I, type II, and type III IFNs. Type I IFN responses are predominantly mediated by IFN-α and IFN-β in a variety of immune cells. The type II IFN family consists of IFN-γ, which is predominantly produced by T and NK cells. The type III IFN responses are induced by IFN-λ early on in viral infections, and this suppresses initial viral dissemination without instigating inflammation [119]. All three IFN families can modulate the immune system and induce an antiviral state in cells.

In the context of COVID-19, there is a paradox regarding IFN responses. Patients with COVID-19 have been reported to show both elevated and reduced levels of IFNs, and severe or critical COVID-19 cases are characterized by aberrant IFN responses [120–122]. Furthermore, polymorphisms in IFN-associated genes, including *IFNAR2*, *OAS1*, and *TYK2*, are associated with critical cases of COVID-19 [123, 124]. Two major underlying factors have been associated with this vulnerability. First, some patients produce autoantibodies against type I IFNs (most prominently to IFNα2 and IFNω) [125–127], and this production increases with age [128, 129]. Second, some patients have genetic ‘inborn errors of immunity’ [29] or loss-of-function mutations in critical genes. These mutations can occur in genes associated with viral RNA sensing and the initiation of IFN production, such as TLRs [29, 31, 33, 39, 40], or in other more downstream genes in the IFN production and signaling pathway, such as TBK1, IRF7, and IFNAR1 [123, 124]. These human genetic and immunological determinants have been suggested to account for up to 20% of all COVID-19 deaths [129].

**Fig. 2:**
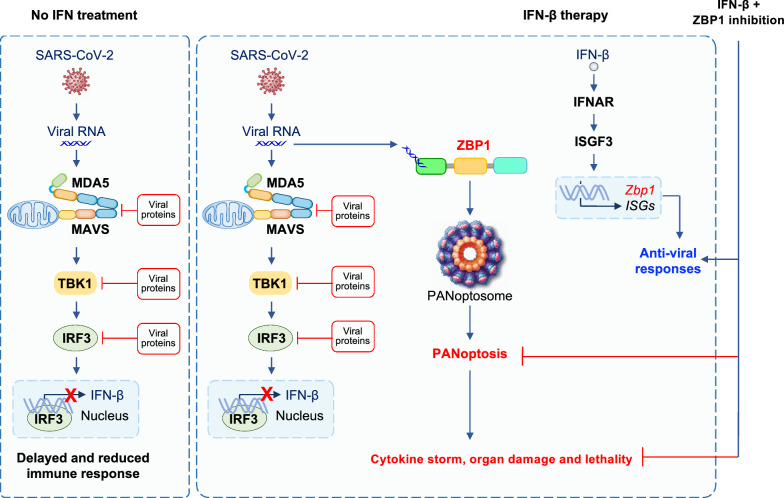
Interferon (IFN) therapy following SARS-CoV-2 infection induces cytokine storm, organ damage, and lethality. SARS-CoV-2 has evolved to evade innate immune sensing mechanisms. Several components from SARS-CoV-2 inhibit type I IFN production by interfering with molecules involved in IFN production such as melanoma differentiation-associated protein 5 (MDA5), mitochondrial antiviral signaling (MAVS), tumor necrosis factor receptor-associated factor (TRAF)-associated NF-κΒ (TANK)-binding kinase 1 (TBK1), and IFN regulatory factor 3 (IRF3). To overcome this, IFN therapy has been suggested to treat patients with COVID-19. However, IFNs induce multiple IFN stimulated genes (ISGs), which can have both anti-viral as well as pro-death functions. Z-DNA binding protein 1 (ZBP1) is one such molecule which senses viral RNA to assemble the ZBP1-PANoptosome, thereby executing PANoptosis to drive cytokine storm, organ damage, and even lethality. This can impact the efficacy of IFN therapy in COVID-19. Strategies to inhibit ZBP1 could improve the efficacy of IFN therapy in patients with COVID-19

The reduction in type I and III IFNs observed in the serum of patients with mild and moderate COVID-19 [120] could possibly be due to the ability of SARS-CoV-2 to evade the immune system. Indeed, SARS-CoV-2 infection limits type I and III IFN production by preventing the release of mRNA from its transcription site and/or triggering its nuclear degradation [130]. Moreover, several proteins from SARS-CoV-2 are known to disrupt RLR and TLR sensing pathways for IFN production (Fig. 2). For example, ISGylation of the caspase activation and recruitment domain (CARD) of MDA5 is crucial for its activation following infection by an RNA virus, but ISG15-mediated ISGylation of MDA5 can be suppressed by the SARS-CoV-2 papain-like protease [131]. Additionally, SARS-CoV-2 ORF9b, N, and M proteins inhibit IFN-β expression by interfering with RIG-I and MDA5 pathways [132–134]. ORF9b also blocks the TLR3-TRIF pathway [135]. Nearly all the SARS-CoV-2 proteins have been suggested to block IFN production and signaling at various points in the IFN pathway, and this has been extensively characterized [136]. Aside from blocking TLR and RLR signaling specifically, ORF6 prevents nuclear localization of the transcription factor signal transducer and activator of transcription 1 (STAT1), which is needed for IFN production [137]. On a whole-cell level, SARS-CoV-2 nonstructural protein (NSP)1 and NSP14 inhibit translation, preventing the expression of components in the IFN signaling pathway [138–140].

To overcome these viral evasion strategies, IFN therapy has been suggested as a potential COVID-19 treatment. Preclinical studies have shown that treatment with exogenous type I IFN or STING or RIG-I agonists, which induce IFN production, can lower the viral load if given prophylactically [81–83, 141]; however, this effect on viral load is limited once infection is established [142]. Additionally, the outcomes of IFN-based therapy have been inconsistent in patients with COVID-19 [143]. Although administration of IFN-α2b in the first five days of hospitalization is associated with reduced mortality, delayed IFN-α2b treatment results in extended hospital stays and slows recovery compared to patients receiving IFN earlier in the course of disease [144]. Furthermore, while Peginterferon lambda 1a, a pegylated recombinant IFN-λ1a, reduces SARS-CoV-2 replication in mice [145], it is ineffective in resolving symptoms and other clinical metrics in patients with mild disease [146]. The WHO Solidarity Trial Consortium clinical trial also found that type I IFN treatment is not beneficial in treating SARS-CoV-2 infection [147]. Studies in influenza infections have shown that type I and type III IFNs inhibit lung epithelial repair [148], and it is possible similar effects also occur in patients with COVID-19. Additionally, mechanistic preclinical studies have suggested that the lack of efficacy for type I IFN therapy is due to IFN-mediated inflammatory responses, cytokine storm, and inflammatory cell death, PANoptosis, that occur in a ZBP1-dependent manner during coronavirus infections (Fig. 2) [13]. IFNs not only induce the expression of ISGs to block viral replication, but they also upregulate cell death mediators such as ZBP1 (Fig. 2), an innate immune sensor known to activate PANoptosis [13, 114–118, 149]. The administration of type I IFN promotes lethality associated with inflammation and PANoptosis in the lungs of mice infected with MHV, while this is prevented in *Zbp1*^–/–^ mice [13]. Similarly, treatment with type I or type II IFNs in human or murine macrophages potentiates PANoptosis during infection with SARS-CoV-2 or MHV, respectively, in a ZBP1-dependent manner [13]. Patients who succumb to COVID-19 infection have higher expression of *ZBP1* in their immune cells compared to non-hospitalized patients with stable COVID-19, further suggesting that there may be a pathological role for ZBP1 in driving COVID-19 severity during IFN therapy [13].

Altogether, the data suggest that IFN-based therapy may improve clinical outcomes in some patients if given early in the course of disease. Therefore, timing and duration are critical parameters of endogenous IFN responses and should be considered carefully for therapeutic strategies against viral infections. Understanding the molecular mechanisms associated with IFN signaling is essential to improve the potential efficacy of IFN therapy in patients with COVID-19.

## TNF and IFN-γ signaling, PANoptosis, and cytokine storm in COVID-19 pathogenesis

**Fig. 3:**
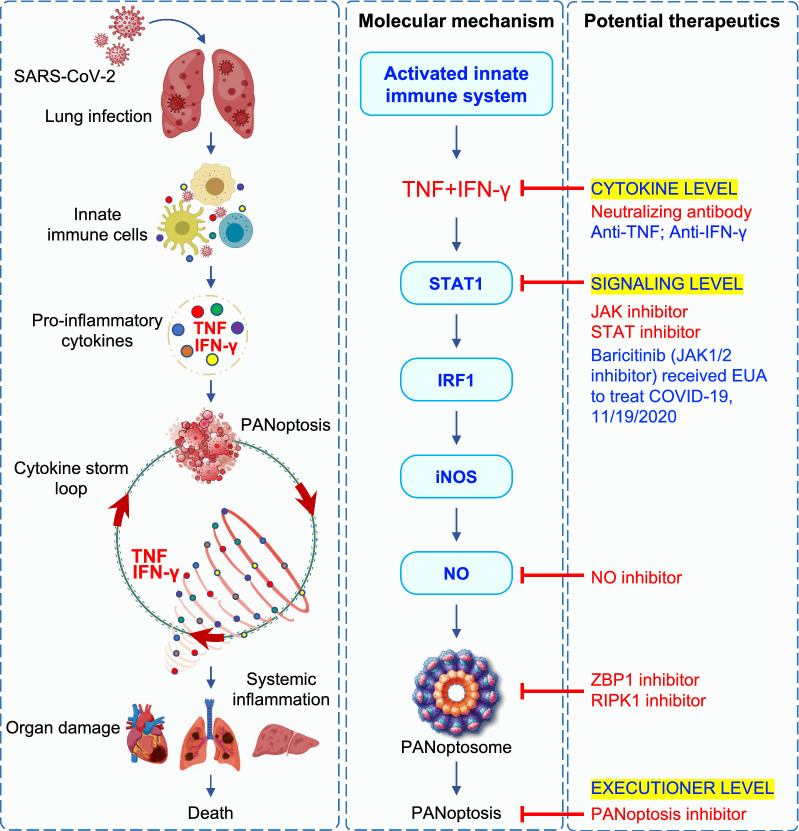
Cytokine storm: Molecular mechanism and potential therapeutics in COVID-19. Lung infection by SARS-CoV-2 leads to production of several pro-inflammatory cytokines by innate immune cells. The combination of tumor necrosis factor (TNF) and interferon (IFN)-γ activates PANoptosis leading to a cytokine storm loop, which further potentiates cytokine release and perpetuates PANoptosis. This positive feedback loop can result in systemic inflammation, multiorgan failure, and lethality. Synergism of TNF and IFN-γ activates signal transducer and activator of transcription 1 (STAT1) signaling and induces expression of IFN regulatory factor 1 (IRF1), which regulates inducible nitric oxide synthase (iNOS) for nitric oxide (NO) production. This pathway leads to the induction of PANoptosis, which is regulated through a multiprotein complex called the PANoptosome. Based on the molecular mechanisms engaged by TNF and IFN-γ for the activation of PANoptosis, several drugs can potentially be repurposed for COVID-19 treatment

In addition to the critical roles of IFNs in responding to SARS-CoV-2 infection, other cytokines released because of innate immune activation have also been implicated. Recent studies have demonstrated a positive feedback loop whereby cytokine release causes PANoptosis to facilitate more cytokine release, spiraling toward a life-threatening cytokine storm that damages host tissues and organs (Fig. 3) [5, 12]. TNF and IFN-γ are suggested to be central to the induction of cytokine storm and pathogenicity. Levels of these cytokines are elevated in the serum of patients with severe COVID-19 [4, 121, 150]. In macrophages, TNF and IFN-γ signal cooperatively to induce PANoptosis [12]. The co-administration of TNF and IFN-γ in mice causes lethal shock [12, 151], with physiological symptoms that are consistent with those seen in patients with severe COVID-19, including multiorgan damage and dysfunction. Excessive cytokine signaling and cell death are likely drivers of these symptoms. For instance, structural damage to cell membranes contributes to vascular leakage and the development of a pro-coagulative endothelium, which initiates and propagates ΑRDS and lung damage in cytokine storm syndromes, including COVID-19 [152]. Vascular leakage or damage is also associated with an unprecedented group of hyper-inflammatory shock syndromes observed in children exposed to COVID-19, referred to as multisystem inflammatory syndrome in children (MIS-C) [153, 154]. Vascular leakage could be caused by endothelial cell damage as a result of the upregulated cytokines, particularly by the synergism of TNF and IFN-γ. In addition, pro-inflammatory cytokines have been shown to damage endothelial cell-associated anticoagulant pathways [155]. Another hallmark of TNF and IFN-γ–shock or COVID-19 is lymphopenia and immunosuppression [156]. Postmortem examination of spleens and lymph nodes from patients with COVID-19 has identified a lack of germinal centers [157], possibly due to cell death of lymphocytes driven by TNF and IFN-γ. Indeed, severe COVID-19 cases have been reported to have high amounts of TNF in the germinal centers, thereby limiting appropriate immune responses [157]. Increased serum alanine transaminase (ALT) and aspartate aminotransferase (AST) observed in patients with COVID-19 could also be due to the action of TNF and IFN-γ in hepatocytes. Indeed, mice deficient in TNF and IFN-γ have reduced ALT and AST in response to Hepatitis B virus surface antigen, suggesting that TNF and IFN-γ together can trigger liver injury [158].

Molecularly, synergism of TNF and IFN-γ engages the janus kinase (JAK)-STAT1 axis to induce IRF1 expression and nitric oxide (NO) production, which activates PANoptosis (Fig. 3) [12], underscoring the importance of these molecules in the pathology of COVID-19 and other cytokine storm syndromes. Indeed, higher levels of IRF1 and inducible nitric oxide synthase (iNOS) in patients with COVID-19 are associated with severe disease and poor clinical outcomes [12]. Moreover, a single cell transcriptional study performed in PBMCs from patients with COVID-19 suggests that IFN-α and IFN-γ function in T cells and DCs to promote disease severity through activating STAT1 [159]. Furthermore, deletion of nitric oxide synthase 2 (*Nos2)* or *Casp8*, another key molecule in PANoptosis, reduces SARS-CoV-2 infection-driven weight loss without impacting peak viral burdens in mice, suggesting there may be a pathogenic role for the iNOS-caspase-8 axis in COVID-19 [160]. Consistently, *Casp8*^–/–^*Ripk3*^–/–^ mice, but not *Ripk3*^–/–^ mice, are resistant to the lethality induced by TNF and IFN-γ shock, extending the regulatory role of caspase-8 in cytokine storm-associated diseases [12]. Moreover, a computational strategy integrating over 300,000 single-cell transcriptomes found that increased levels of ‘inflammatory macrophages’ are associated with an inflammatory state and disease severity in patients with COVID-19 as well as in those with autoimmune diseases like rheumatoid arthritis, and inflammatory diseases like Crohn’s disease and ulcerative colitis [161]. The overall transcriptome program of patient-derived ‘inflammatory macrophages’ closely aligns with that of macrophages stimulated by the combination of TNF and IFN-γ, and these cells are distinguished by high levels of *STAT1*, *IFNGR1*, *IFNGR2*, *NFKB1*, *IL1B*, and other molecules [161]. Together, these results support the role of TNF and IFN-γ synergism in driving COVID-19 disease progression in cytokine storm syndromes.

## Key clinical examples of SARS-CoV-2 immunopathology

The pathogenesis of SARS-CoV-2–induced pneumonia has been suggested to occur through both virus-mediated tissue damage and inflammation-mediated damage [162]. Inflammation-mediated damage occurs when effector immune cells are recruited and cause local and systemic inflammatory responses that may even persist after the virus is cleared. Autopsies of deceased patients with COVID-19 have identified substantial accumulation of activated immune cells in response to a small viral load [163, 164], indicating that tissue damage and organ failure may be caused by an overactivated immune system instead of virus-mediated tissue damage. When viral clearance is delayed due to viral immune evasion or a weak activation of the innate immune system, the levels of circulating immature myeloid cells can increase, producing an abundance of inflammatory mediators to increase vascular permeability and organ damage [163, 164]. Compromised lung function in patients with COVID-19 is associated with excess vascular permeability which can cause microthrombi to form [165] in addition to a range of systemic symptoms. By contrast, tissue resident myeloid cells in lungs are depleted in patients with severe COVID-19 [166, 167] possibly due to excessive cell death either as a result of the virus or from excess inflammation. The pathogenesis of extrapulmonary manifestations such as olfactory dysfunction [168], gastrointestinal symptoms [169], and cardiac and renal dysfunction [165] can be caused by multiple events including direct viral injury, cytokine-mediated damage, auto-antibody induced tissue damage, or vascular damage. Hypercoagulation, endothelial damage, and thromboembolism are common in patients with severe COVID-19 [169], and may come from direct viral damage to the vasculature or severe inflammatory responses. The altered vascular endothelial homeostasis induces the activation of monocytes, platelets, and macrophages thereby promoting the release of factor VIII, von Willebrand factor, and tissue factor, which leads to the production of thrombin and fibrin clot formation [165, 170].

## Immune-directed therapy for COVID-19

Vaccination has served as the major strategy to attempt to bring an end to the COVID-19 pandemic, and a variety of SARS-CoV-2 S protein-targeting vaccines have been produced, including those which utilize lipid nanoparticle-encapsulated mRNA, inactivated virions, or viral-vectors. These vaccines have reduced infection rates, hospitalizations, and deaths. However, mutations in evolving variants including B.1.351 (Beta), B.1.1.28 (Gamma), B.1.617.2 (Delta), and B.1.1.529 (Omicron) facilitate immune-escape by rendering the viruses less susceptible to immunity from either vaccines or from previous infections, as these variants carry mutations that reduce the affinity of antibody binding [171]. Although the variants such as B.1.1.7 (Alpha) and B.1.617.2 (Delta) are known to spread more efficiently than others, the association of these variants with the severity of COVID-19 is less apparent, and whether they also alter innate immune detection remains to be fully characterized. Therefore, as new variants emerge, it is important to continue working to understand their ability to immune-escape and induce immune responses.

In addition to vaccination, agents such as antivirals and immunomodulators have been investigated, and some are approved by the FDA for the treatment of patients with COVID-19. For example, the viral polymerase inhibitor remdesivir received FDA approval for use in hospitalized patients. Moreover, the FDA has issued emergency use authorization (EUA) for antiviral drugs such as molnupiravir and ritonavir-boosted nirmatrelvir (Paxlovid), immunomodulators such as baricitinib (JAK inhibitor) and anakinra (IL-1 receptor antagonist), and monoclonal antibodies such as tocilizumab (an IL-6 receptor monoclonal antibody) [172]. However, many of these drugs have limited therapeutic efficacy, leaving many cases of severe disease without effective treatments.

To manage patients with severe COVID-19, many clinical therapies have been repurposed. As discussed above, administration of IFN has also been considered as a therapeutic strategy, with mixed results [143–146], and preclinical studies have suggested potential benefits from treatment with STING or RIG-I agonists to induce IFN production [81–83, 141], though STING inhibitors have also shown promise [77]. There are likely timing-dependent effects for these treatments. In addition, immunomodulatory agents have been studied for their efficacy against COVID-19. Corticosteroids, such as dexamethasone, work by suppressing a broad range of immune responses, including inflammation. However, clinical trials found that dexamethasone only reduces mortality in a subset of patients with COVID-19, specifically hospitalized patients requiring mechanical ventilation or supplemental oxygen [173]. Additionally, corticosteroids inhibit immune responses systemically, leading to an increased risk of secondary infections. Therefore, a targeted approach to modulate specific aspects of the immune response may be a more suitable option. Since elevated IL-6 levels are associated with COVID-19 severity [121, 150], blocking IL-6 signaling has been proposed for treating patients with COVID-19. However, blocking IL-6 signaling has shown varied responses. Tocilizumab, an anti–IL-6 receptor-blocking antibody, has received EUA from the FDA after showing efficacy in hospitalized patients requiring supplemental oxygen [174]. However, phase 3 COVACTA and REMDACTA (tocilizumab) and Kevzara and SARTRE (sarilumab) trials show that anti–IL-6 therapies fail to improve clinical outcomes or reduce mortality [175–178]. Since the inflammasome-dependent cytokine IL-1β is also elevated in patients with COVID-19 [179, 180], drugs that block IL-1 signaling can potentially interrupt inflammatory responses and have been tested. The anti–IL-1β antibody, canakinumab, has improved clinical symptoms in hospitalized patients with COVID-19 in some studies [181], but was not found to improve survival when compared to control groups [182]. In contrast, patients who received anakinra, an IL-1 receptor antagonist, have shown reduced inflammatory markers [183] and mortality [184, 185], leading to anakinra receiving EUA for the treatment of hospitalized patients on supplemental oxygen. Upstream of IL-1β production, inhibition of the NLRP3 inflammasome has also been considered as a potential immunomodulatory strategy. The NLRP3 inhibitors MCC950 and glyburide reduce release of IL-1β and IL-6, respectively, in SARS-CoV-2–infected human monocytes, suggesting they have potential therapeutic efficacy. A small, randomized trial of patients with COVID-19 demonstrated the ability of colchicine, which indirectly inhibits the NLRP3 inflammasome, to reduce the requirement of oxygen [186], though current NIH guidelines do not recommend colchicine for the treatment of COVID-19 [187]. In addition, both TNF and IFN-γ are elevated in patients with COVID-19, and preclinical studies have suggested combined inhibition of TNF and IFN-γ signaling can decrease the effects of cytokine storm driven by PANoptosis (Fig. 3) [5, 12, 160]. Case studies have suggested that patients on anti-TNF therapy for rheumatic diseases tend to have lower hospitalization rates from COVID-19 [188], suggesting a prophylactic effect of anti-TNF therapy in the disease pathogenesis. Furthermore, the JAK-STAT signaling pathway that induces PANoptosis in response to TNF and IFN-γ can be targeted to suppress inflammation and improve clinical symptoms (Fig. 3) [12]. Indeed, baricitinib, a JAK1-JAK2 inhibitor, received EUA for the treatment of COVID-19 in combination with remdesivir based on its efficacy in reducing recovery time in hospitalized patients [189].

Overall, there are conflicting findings and mixed responses regarding the use of immune-directed therapy for COVID-19. Therefore, expanded approaches, such as targeting multiple cytokines concurrently, with or without antiviral treatments or antibodies, should continue to be evaluated. Additionally, the stage of disease development should also be considered when postulating immunomodulatory therapies. Blocking inflammatory signaling in specific phases of disease development may be beneficial, while this approach may be detrimental at other times.

## Summary and future directions

SARS-CoV-2 and COVID-19 continue to cause an unprecedented global social, economic, and public health burden despite the many advances in our understanding of the basic and translational science underlying the infection. Continued efforts to elucidate the cellular and molecular basis of SARS-CoV-2 infection and the associated immunopathology that leads to the development of COVID-19 are needed to advance treatment strategies and prevention.

Although we are now starting to understand the role of innate immunity, PANoptosis, and cytokine storm as they relate to COVID-19 pathogenesis, several questions remain. For instance, why are innate immune pathways dysregulated in some individuals but not in others during SARS-CoV-2 infection? The magnitude and duration of immune responses may vary between individuals due to inherent genetic or immunological traits. Considering the importance of innate immune signaling in providing protection against SARS-CoV-2 infection, genetic heterogeneity (polymorphisms) in the molecules involved in innate immunity may explain why some patients infected with SARS-CoV-2 are pauci-symptomatic and others develop severe disease. Moreover, age, gender, hormones, and underlying diseases and comorbidities significantly affect COVID-19 outcomes. Therefore, we will likely gain insight into spatiotemporal patterns of immune responses associated with each patient’s prognosis or clinical outcome by performing rigorous genetic testing and immunological phenotyping of those who contract the virus but do not develop severe clinical symptoms. Additionally, dynamic and longitudinal monitoring of innate immune responses in patients with COVID-19 could provide further clarity. Some patients who recover from COVID-19 experience long-term health effects for four or more weeks, which is often referred to as Long Covid syndrome or post-acute sequelae of SARS-CoV-2 infection (PASC) [190]. For some, continuous symptoms last for weeks, while others have a recurrence of symptoms, and some develop completely new symptoms. The immunobiology of PASC remains under investigation. However, leading hypotheses include (i) chronic inflammation mediated by persistent virus or viral RNA and antigens in the tissues, (ii) production of autoantibodies, (iii) microbial dysbiosis, and (iv) defects in tissue repair [191]. Moreover, dysregulated innate immune stimulation is associated with PASC [190]. Further work on PASC is needed to better understand the underlying molecular mechanisms to determine effective treatment strategies.

SARS-CoV-2 continuously evolves through mutations in the genetic code during replication. The WHO defines a SARS-CoV-2 variant of concern as one that is more likely to cause infections even in those who are vaccinated or in those who were previously infected. The pathology caused by SARS-CoV-2 variants depends on the infectivity and transmissibility of the variants, and prediction of SARS-CoV-2’s molecular evolution is extremely challenging. Therefore, to counter these infections, strategies that target host pathways rather than viral proteins may be advantageous. Therapies that have shown preclinical promise, such as targeting cytokines and cytokine receptors, are entering clinical trials. In addition, targeting PANoptosis and the host cell death machinery to suppress cytokine storm can be considered for mitigating severe COVID-19. Since PANoptosis mediated by ZBP1 impedes the therapeutic efficacy of IFN treatment [13], molecules that target ZBP1 activation could also be designed. Molecularly, ADAR1 has been shown to suppress ZBP1-mediated PANoptosis [109], but the role of ADAR1 in COVID-19 remains unknown. Understanding these functional relationships in the context of SARS-CoV-2 infection and COVID-19 will be critical for identifying new therapeutic strategies.

In addition to identifying innate immune pathways and molecular targets to counteract inflammatory pathology in COVID-19, identifying the clinically appropriate time to intervene is also critical. In the early stage of infection, inflammatory responses are crucial to clear pathogens, and intervening too early may interfere with the development of effective immunity. However, late interventions may not control the overt inflammatory responses, leading to dangerous consequences. Determining the optimal timing of treatment is further complicated because most patients only present at the hospital when they develop severe clinical symptoms. Therefore, proper patient stratification and timing of treatment are important to consider at presentation to determine whether targeting innate immunity and inflammatory cell death may be beneficial.

Furthermore, with the molecular redundancies encoded within the key molecules of the innate immune system, it is also possible that pharmaceutical therapies that target single pathways may not be effective at dampening inflammation and pathology. The specific mechanisms underlying how cells detect viruses and how cell death is executed may also be tissue- and cell-specific. Though many studies have shown that a core set of cytokines are associated with severe COVID-19, there is still much to learn about the cellular mechanisms influencing their release. More controlled clinical and pre-clinical studies are needed to understand overall molecular mechanisms of COVID-19 pathogenesis and to identify therapeutic targets.

## Data Availability

Not applicable.

## References

[1] Tan W, Zhao X, Ma X, Wang W, Niu P, Xu W (2020). A novel coronavirus genome identified in a cluster of pneumonia cases—Wuhan, China 2019–2020. China CDC Wkly.

[2] Cucinotta D, Vanelli M (2020). WHO declares COVID-19 a pandemic. Acta Biomed.

[3] Dong E, Du H, Gardner L (2020). An interactive web-based dashboard to track COVID-19 in real time. Lancet Infect Dis.

[4] Huang C, Wang Y, Li X, Ren L, Zhao J, Hu Y (2020). Clinical features of patients infected with 2019 novel coronavirus in Wuhan. China Lancet.

[5] Karki R, Kanneganti TD (2021). The 'cytokine storm': molecular mechanisms and therapeutic prospects. Trends Immunol.

[6] Zheng Z, Peng F, Xu B, Zhao J, Liu H, Peng J (2020). Risk factors of critical & mortal COVID-19 cases: a systematic literature review and meta-analysis. J Infect.

[7] Karki R, Kanneganti TD (2019). Diverging inflammasome signals in tumorigenesis and potential targeting. Nat Rev Cancer.

[8] Guo C, Li B, Ma H, Wang X, Cai P, Yu Q (2020). Single-cell analysis of two severe COVID-19 patients reveals a monocyte-associated and tocilizumab-responding cytokine storm. Nat Commun.

[9] Qin G, Liu S, Yang L, Yu W, Zhang Y (2021). Myeloid cells in COVID-19 microenvironment. Signal Transduct Target Ther.

[10] Park A, Iwasaki A (2020). Type I and type III interferons—induction, signaling, evasion, and application to combat COVID-19. Cell Host Microbe.

[11] Vabret N, Britton GJ, Gruber C, Hegde S, Kim J, Kuksin M (2020). Immunology of COVID-19: current state of the science. Immunity.

[12] Karki R, Sharma BR, Tuladhar S, Williams EP, Zalduondo L, Samir P (2021). Synergism of TNF-alpha and IFN-gamma triggers inflammatory cell death, tissue damage, and mortality in SARS-CoV-2 infection and cytokine shock syndromes. Cell.

[13] Karki R, Lee S, Mall R, Pandian N, Wang Y, Sharma BR (2022). ZBP1-dependent inflammatory cell death, PANoptosis, and cytokine storm disrupt IFN therapeutic efficacy during coronavirus infection. Sci Immunol..

[14] Diamond MS, Kanneganti TD (2022). Innate immunity: the first line of defense against SARS-CoV-2. Nat Immunol.

[15] Liu G, Zhao Y (2007). Toll-like receptors and immune regulation: their direct and indirect modulation on regulatory CD4+ CD25+ T cells. Immunology.

[16] Akira S, Takeda K (2004). Toll-like receptor signalling. Nat Rev Immunol.

[17] Thompson CM, Holden TD, Rona G, Laxmanan B, Black RA, Keefe GE (2014). Toll-like receptor 1 polymorphisms and associated outcomes in sepsis after traumatic injury: a candidate gene association study. Ann Surg.

[18] Zheng M, Karki R, Williams EP, Yang D, Fitzpatrick E, Vogel P (2021). TLR2 senses the SARS-CoV-2 envelope protein to produce inflammatory cytokines. Nat Immunol.

[19] Jung S, Potapov I, Chillara S, Del Sol A (2021). Leveraging systems biology for predicting modulators of inflammation in patients with COVID-19. Sci Adv.

[20] van der Sluis RM, Cham LB, Gris-Oliver A, Gammelgaard KR, Pedersen JG, Idorn M (2022). TLR2 and TLR7 mediate distinct immunopathological and antiviral plasmacytoid dendritic cell responses to SARS-CoV-2 infection. EMBO J.

[21] Proud PC, Tsitoura D, Watson RJ, Chua BY, Aram MJ, Bewley KR (2021). Prophylactic intranasal administration of a TLR2/6 agonist reduces upper respiratory tract viral shedding in a SARS-CoV-2 challenge ferret model. EBioMedicine.

[22] Lee S, Channappanavar R, Kanneganti TD (2020). Coronaviruses: Innate immunity, inflammasome activation, inflammatory cell death, and cytokines. Trends Immunol.

[23] Zhao J, Wohlford-Lenane C, Zhao J, Fleming E, Lane TE, McCray PB (2012). Intranasal treatment with poly(I•C) protects aged mice from lethal respiratory virus infections. J Virol.

[24] Barnard DL, Day CW, Bailey K, Heiner M, Montgomery R, Lauridsen L (2006). Evaluation of immunomodulators, interferons and known in vitro SARS-coV inhibitors for inhibition of SARS-coV replication in BALB/c mice. Antivir Chem Chemother.

[25] Totura AL, Whitmore A, Agnihothram S, Schäfer A, Katze MG, Heise MT (2015). Toll-like receptor 3 signaling via TRIF contributes to a protective innate immune response to severe acute respiratory syndrome coronavirus infection. mBio.

[26] Bortolotti D, Gentili V, Rizzo S, Schiuma G, Beltrami S, Strazzabosco G (2021). TLR3 and TLR7 RNA sensor activation during SARS-CoV-2 infection. Microorganisms..

[27] Tamir H, Melamed S, Erez N, Politi B, Yahalom-Ronen Y, Achdout H (2022). Induction of innate immune response by TLR3 agonist protects mice against SARS-CoV-2 infection. Viruses.

[28] Menezes MC, Veiga ADM, Martins de Lima T, Kunimi Kubo Ariga S, Vieira Barbeiro H, de Moreira Lucena C (2011). Lower peripheral blood Toll-like receptor 3 expression is associated with an unfavorable outcome in severe COVID-19 patients. Sci Rep.

[29] Zhang Q, Bastard P, Liu Z, Le Pen J, Moncada-Velez M, Chen J (2020). Inborn errors of type I IFN immunity in patients with life-threatening COVID-19. Science.

[30] Povysil G, Butler-Laporte G, Shang N, Wang C, Khan A, Alaamery M (2021). Rare loss-of-function variants in type I IFN immunity genes are not associated with severe COVID-19. J Clin Invest.

[31] Alseoudy MM, Elgamal M, Abdelghany DA, Borg AM, El-Mesery A, Elzeiny D (2022). Prognostic impact of toll-like receptors gene polymorphism on outcome of COVID-19 pneumonia: a case-control study. Clin Immunol.

[32] Choudhury A, Mukherjee S (2020). In silico studies on the comparative characterization of the interactions of SARS-CoV-2 spike glycoprotein with ACE-2 receptor homologs and human TLRs. J Med Virol.

[33] Taha SI, Shata AK, Baioumy SA, Fouad SH, Anis SG, Mossad IM (2021). Toll-like receptor 4 polymorphisms (896A/G and 1196C/T) as an indicator of COVID-19 severity in a convenience sample of Egyptian patients. J Inflamm Res.

[34] Zhao Y, Kuang M, Li J, Zhu L, Jia Z, Guo X (2021). SARS-CoV-2 spike protein interacts with and activates TLR41. Cell Res.

[35] Petruk G, Puthia M, Petrlova J, Samsudin F, Strömdahl AC, Cerps S (2020). SARS-CoV-2 spike protein binds to bacterial lipopolysaccharide and boosts proinflammatory activity. J Mol Cell Biol.

[36] Schön MP, Schön M (2008). TLR7 and TLR8 as targets in cancer therapy. Oncogene.

[37] Moreno-Eutimio MA, López-Macías C, Pastelin-Palacios R (2020). Bioinformatic analysis and identification of single-stranded RNA sequences recognized by TLR7/8 in the SARS-CoV-2, SARS-CoV, and MERS-CoV genomes. Microbes Infect.

[38] Melchjorsen J, Jensen SB, Malmgaard L, Rasmussen SB, Weber F, Bowie AG (2005). Activation of innate defense against a paramyxovirus is mediated by RIG-I and TLR7 and TLR8 in a cell-type-specific manner. J Virol.

[39] Asano T, Boisson B, Onodi F, Matuozzo D, Moncada-Velez M, Maglorius Renkilaraj MRL (2021). X-linked recessive TLR7 deficiency in ~1% of men under 60 years old with life-threatening COVID-19. Sci Immunol..

[40] Abolhassani H, Vosughimotlagh A, Asano T, Landegren N, Boisson B, Delavari S (2022). X-Linked TLR7 deficiency underlies critical COVID-19 pneumonia in a male patient with ataxia-telangiectasia. J Clin Immunol.

[41] van der Made CI, Simons A, Schuurs-Hoeijmakers J, van den Heuvel G, Mantere T, Kersten S (2020). Presence of genetic variants among young men with severe COVID-19. JAMA.

[42] Klein SL, Dhakal S, Ursin RL, Deshpande S, Sandberg K, Mauvais-Jarvis F (2020). Biological sex impacts COVID-19 outcomes. PLoS Pathog.

[43] Hurst J, Prinz N, Lorenz M, Bauer S, Chapman J, Lackner KJ (2009). TLR7 and TLR8 ligands and antiphospholipid antibodies show synergistic effects on the induction of IL-1beta and caspase-1 in monocytes and dendritic cells. Immunobiology.

[44] Döring Y, Hurst J, Lorenz M, Prinz N, Clemens N, Drechsler MD (2010). Human antiphospholipid antibodies induce TNFalpha in monocytes via Toll-like receptor 8. Immunobiology.

[45] Amezcua-Guerra LM, Rojas-Velasco G, Brianza-Padilla M, Vázquez-Rangel A, Márquez-Velasco R, Baranda-Tovar F (2020). Presence of antiphospholipid antibodies in COVID-19: case series study. Ann Rheum Dis.

[46] Borghi MO, Beltagy A, Garrafa E, Curreli D, Cecchini G, Bodio C (2020). Anti-phospholipid antibodies in COVID-19 are different from those detectable in the anti-phospholipid syndrome. Front Immunol.

[47] Yin X, Riva L, Pu Y, Martin-Sancho L, Kanamune J, Yamamoto Y (2021). MDA5 governs the innate immune response to SARS-CoV-2 in lung epithelial cells. Cell Rep.

[48] Yang DM, Geng TT, Harrison AG, Wang PH (2021). Differential roles of RIG-I like receptors in SARS-CoV-2 infection. Mil Med Res.

[49] Loo YM, Gale M (2011). Immune signaling by RIG-I-like receptors. Immunity.

[50] Horner SM, Liu HM, Park HS, Briley J, Gale M (2011). Mitochondrial-associated endoplasmic reticulum membranes (MAM) form innate immune synapses and are targeted by hepatitis C virus. Proc Natl Acad Sci U S A.

[51] Thorne LG, Reuschl AK, Zuliani-Alvarez L, Whelan MVX, Turner J, Noursadeghi M (2021). SARS-CoV-2 sensing by RIG-I and MDA5 links epithelial infection to macrophage inflammation. EMBO J.

[52] Loske J, Röhmel J, Lukassen S, Stricker S, Magalhães VG, Liebig J (2022). Pre-activated antiviral innate immunity in the upper airways controls early SARS-CoV-2 infection in children. Nat Biotech.

[53] Kouwaki T, Nishimura T, Wang G, Oshiumi H (2021). RIG-I-like receptor-mediated recognition of viral genomic RNA of severe acute respiratory syndrome coronavirus-2 and viral escape from the host innate immune responses. Front Immunol.

[54] Yamada T, Sato S, Sotoyama Y, Orba Y, Sawa H, Yamauchi H (2021). RIG-I triggers a signaling-abortive anti-SARS-CoV-2 defense in human lung cells. Nat Immunol.

[55] Wu XM, Zhang J, Li PW, Hu YW, Cao L, Ouyang S (2020). NOD1 promotes antiviral signaling by binding viral RNA and regulating the interaction of MDA5 and MAVS. J Immunol.

[56] Christgen S, Kanneganti TD (2020). Inflammasomes and the fine line between defense and disease. Curr Opin Immunol.

[57] Ma J, Zhu F, Zhao M, Shao F, Yu D, Ma J (2021). SARS-CoV-2 nucleocapsid suppresses host pyroptosis by blocking Gasdermin D cleavage. EMBO J.

[58] Zheng M, Williams EP, Malireddi RKS, Karki R, Banoth B, Burton A (2020). Impaired NLRP3 inflammasome activation/pyroptosis leads to robust inflammatory cell death via caspase-8/RIPK3 during coronavirus infection. J Biol Chem.

[59] Rodrigues TS, de Sá KSG, Ishimoto AY, Becerra A, Oliveira S, Almeida L (2021). Inflammasomes are activated in response to SARS-CoV-2 infection and are associated with COVID-19 severity in patients. J Exp Med.

[60] Ferreira AC, Soares VC, de Azevedo-Quintanilha IG, Dias S, Fintelman-Rodrigues N, Sacramento CQ (2021). SARS-CoV-2 engages inflammasome and pyroptosis in human primary monocytes. Cell Death Discov.

[61] Campbell GR, To RK, Hanna J, Spector SA (2021). SARS-CoV-2, SARS-CoV-1, and HIV-1 derived ssRNA sequences activate the NLRP3 inflammasome in human macrophages through a non-classical pathway. iScience..

[62] Pan P, Shen M, Yu Z, Ge W, Chen K, Tian M (2021). SARS-CoV-2 N protein promotes NLRP3 inflammasome activation to induce hyperinflammation. Nat Commun.

[63] Xu H, Akinyemi IA, Chitre SA, Loeb JC, Lednicky JA, McIntosh MT (2022). SARS-CoV-2 viroporin encoded by ORF3a triggers the NLRP3 inflammatory pathway. Virology.

[64] Theobald SJ, Simonis A, Georgomanolis T, Kreer C, Zehner M, Eisfeld HS (2021). Long-lived macrophage reprogramming drives spike protein-mediated inflammasome activation in COVID-19. EMBO Mol Med.

[65] Zeng J, Xie X, Feng XL, Xu L, Han JB, Yu D (2022). Specific inhibition of the NLRP3 inflammasome suppresses immune overactivation and alleviates COVID-19 like pathology in mice. EBioMedicine.

[66] Eltobgy MM, Zani A, Kenney AD, Estfanous S, Kim E, Badr A (2022). Caspase-4/11 exacerbates disease severity in SARS-CoV-2 infection by promoting inflammation and immunothrombosis. Proc Natl Acad Sci U S A.

[67] Zanoni I, Tan Y, Di Gioia M, Broggi A, Ruan J, Shi J (2016). An endogenous caspase-11 ligand elicits interleukin-1 release from living dendritic cells. Science.

[68] Song JW, Lam SM, Fan X, Cao WJ, Wang SY, Tian H (2020). Omics-driven systems interrogation of metabolic dysregulation in COVID-19 pathogenesis. Cell Metab.

[69] Akpınar S, Oran M, Doğan M, Çelikkol A, Erdem I, Turgut B (2021). The role of oxidized phospholipids in COVID-19-associated hypercoagulopathy. Eur Rev Med Pharmacol Sci.

[70] Junqueira C, Crespo Â, Ranjbar S, de Lacerda LB, Lewandrowski M, Ingber J (2022). FcγR-mediated SARS-CoV-2 infection of monocytes activates inflammation. Nature.

[71] Spel L, Martinon F (2021). Detection of viruses by inflammasomes. Curr Opin Virol.

[72] Rogers C, Erkes DA, Nardone A, Aplin AE, Fernandes-Alnemri T, Alnemri ES (2019). Gasdermin pores permeabilize mitochondria to augment caspase-3 activation during apoptosis and inflammasome activation. Nat Commun.

[73] Franz KM, Neidermyer WJ, Tan YJ, Whelan SPJ, Kagan JC (2018). STING-dependent translation inhibition restricts RNA virus replication. Proc Natl Acad Sci U S A.

[74] Sun B, Sundström KB, Chew JJ, Bist P, Gan ES, Tan HC (2017). Dengue virus activates cGAS through the release of mitochondrial DNA. Sci Rep.

[75] Schoggins JW, MacDuff DA, Imanaka N, Gainey MD, Shrestha B, Eitson JL (2014). Pan-viral specificity of IFN-induced genes reveals new roles for cGAS in innate immunity. Nature.

[76] Sun L, Wu J, Du F, Chen X, Chen ZJ (2013). Cyclic GMP-AMP synthase is a cytosolic DNA sensor that activates the type I interferon pathway. Science.

[77] Domizio JD, Gulen MF, Saidoune F, Thacker VV, Yatim A, Sharma K (2022). The cGAS-STING pathway drives type I IFN immunopathology in COVID-19. Nature.

[78] Liu Y, Jesus AA, Marrero B, Yang D, Ramsey SE, Sanchez GAM (2014). Activated STING in a vascular and pulmonary syndrome. N Engl J Med.

[79] Prantner D, Perkins DJ, Lai W, Williams MS, Sharma S, Fitzgerald KA (2012). 5,6-Dimethylxanthenone-4-acetic acid (DMXAA) activates stimulator of interferon gene (STING)-dependent innate immune pathways and is regulated by mitochondrial membrane potential. J Biol Chem.

[80] Neufeldt CJ, Cerikan B, Cortese M, Frankish J, Lee JY, Plociennikowska A (2022). SARS-CoV-2 infection induces a pro-inflammatory cytokine response through cGAS-STING and NF-κB. Commun Biol.

[81] Li M, Ferretti M, Ying B, Descamps H, Lee E, Dittmar M (2021). Pharmacological activation of STING blocks SARS-CoV-2 infection. Sci Immunol..

[82] Humphries F, Shmuel-Galia L, Jiang Z, Wilson R, Landis P, Ng SL (2021). A diamidobenzimidazole STING agonist protects against SARS-CoV-2 infection. Sci Immunol..

[83] Liu W, Reyes HM, Yang JF, Li Y, Stewart KM, Basil MC (2021). Activation of STING signaling pathway effectively blocks human coronavirus infection. J Virol.

[84] Messaoud-Nacer Y, Culerier E, Rose S, Maillet I, Rouxel N, Briault S (2022). STING agonist diABZI induces PANoptosis and DNA mediated acute respiratory distress syndrome (ARDS). Cell Death Dis.

[85] Hoving JC, Wilson GJ, Brown GD (2014). Signalling C-type lectin receptors, microbial recognition and immunity. Cell Microbiol.

[86] Geijtenbeek TBH, Gringhuis SI (2009). Signalling through C-type lectin receptors: shaping immune responses. Nat Rev Immunol.

[87] Lu Q, Liu J, Zhao S, Gomez Castro MF, Laurent-Rolle M, Dong J (2021). SARS-CoV-2 exacerbates proinflammatory responses in myeloid cells through C-type lectin receptors and Tweety family member 2. Immunity.

[88] Thépaut M, Luczkowiak J, Vivès C, Labiod N, Bally I, Lasala F (2021). DC/L-SIGN recognition of spike glycoprotein promotes SARS-CoV-2 trans-infection and can be inhibited by a glycomimetic antagonist. PLoS Pathog.

[89] Amraei R, Yin W, Napoleon MA, Suder EL, Berrigan J, Zhao Q (2021). CD209L/L-SIGN and CD209/DC-SIGN act as receptors for SARS-CoV-2. ACS Cent Sci.

[90] Alvarez CP, Lasala F, Carrillo J, Muñiz O, Corbí AL, Delgado R (2002). C-type lectins DC-SIGN and L-SIGN mediate cellular entry by Ebola virus in cis and in trans. J Virol.

[91] Carbaugh DL, Baric RS, Lazear HM (2019). Envelope protein glycosylation mediates Zika virus pathogenesis. J Virol.

[92] Navarro-Sanchez E, Altmeyer R, Amara A, Schwartz O, Fieschi F, Virelizier JL (2003). Dendritic-cell-specific ICAM3-grabbing non-integrin is essential for the productive infection of human dendritic cells by mosquito-cell-derived dengue viruses. EMBO Rep.

[93] Marzi A, Gramberg T, Simmons G, Möller P, Rennekamp AJ, Krumbiegel M (2004). DC-SIGN and DC-SIGNR interact with the glycoprotein of Marburg virus and the S protein of severe acute respiratory syndrome coronavirus. J Virol.

[94] Gao C, Zeng J, Jia N, Stavenhagen K, Matsumoto Y, Zhang H (2020). SARS-CoV-2 spike protein interacts with multiple innate immune receptors. bioRxiv.

[95] Katz DH, Tahir UA, Ngo D, Benson MD, Bick AG, Pampana A (2020). Proteomic profiling in biracial cohorts implicates DC-SIGN as a mediator of genetic risk in COVID-19. medRxiv..

[96] Varga Z, Flammer AJ, Steiger P, Haberecker M, Andermatt R, Zinkernagel AS (2020). Endothelial cell infection and endotheliitis in COVID-19. Lancet.

[97] Sanders DW, Jumper CC, Ackerman PJ, Bracha D, Donlic A, Kim H (2021). SARS-CoV-2 requires cholesterol for viral entry and pathological syncytia formation. Elife.

[98] Asarnow D, Wang B, Lee WH, Hu Y, Huang CW, Faust B (2021). Structural insight into SARS-CoV-2 neutralizing antibodies and modulation of syncytia. Cell.

[99] Stadlmann S, Hein-Kuhnt R, Singer G (2020). Viropathic multinuclear syncytial giant cells in bronchial fluid from a patient with COVID-19. J Clin Pathol.

[100] Buchrieser J, Dufloo J, Hubert M, Monel B, Planas D, Rajah MM (2020). Syncytia formation by SARS-CoV-2-infected cells. EMBO J.

[101] Braga L, Ali H, Secco I, Chiavacci E, Neves G, Goldhill D (2021). Drugs that inhibit TMEM16 proteins block SARS-CoV-2 spike-induced syncytia. Nature.

[102] Ma H, Zhu Z, Lin H, Wang S, Zhang P, Li Y (2021). Pyroptosis of syncytia formed by fusion of SARS-CoV-2 spike and ACE2-expressing cells. Cell Discov.

[103] Zhang Z, Zheng Y, Niu Z, Zhang B, Wang C, Yao X (2021). SARS-CoV-2 spike protein dictates syncytium-mediated lymphocyte elimination. Cell Death Differ.

[104] Chakraborty S, Basu A (2020). The COVID-19 pandemic: catching up with the cataclysm. F1000Res.

[105] Ren Y, Shu T, Wu D, Mu J, Wang C, Huang M (2020). The ORF3a protein of SARS-CoV-2 induces apoptosis in cells. Cell Mol Immunol.

[106] Li S, Zhang Y, Guan Z, Li H, Ye M, Chen X (2020). SARS-CoV-2 triggers inflammatory responses and cell death through caspase-8 activation. Signal Transduct Target Ther.

[107] Li S, Jiang L, Li X, Lin F, Wang Y, Li B (2020). Clinical and pathological investigation of patients with severe COVID-19. JCI insight.

[108] Malireddi RKS, Karki R, Sundaram B, Kancharana B, Lee S, Samir P (2021). Inflammatory cell death, PANoptosis, mediated by cytokines in diverse cancer lineages inhibits tumor growth. Immunohorizons..

[109] Karki R, Sundaram B, Sharma BR, Lee S, Malireddi RKS, Nguyen LN (2021). ADAR1 restricts ZBP1-mediated immune response and PANoptosis to promote tumorigenesis. Cell Rep.

[110] Malireddi RKS, Gurung P, Kesavardhana S, Samir P, Burton A, Mummareddy H, Vogel P, Pelletier S, Burgula S, Kanneganti TD (2020). Innate immune priming in the absence of TAK1 drives RIPK1 kinase activity-independent pyroptosis, apoptosis, necroptosis, and inflammatory disease. J Exp Med.

[111] Lukens JR, Gurung P, Vogel P, Johnson GR, Carter RA, McGoldrick DJ (2014). Dietary modulation of the microbiome affects autoinflammatory disease. Nature.

[112] Karki R, Sharma BR, Lee E, Banoth B, Malireddi RKS, Samir P (2020). Interferon regulatory factor 1 regulates PANoptosis to prevent colorectal cancer. JCI insight.

[113] Christgen S, Zheng M, Kesavardhana S, Karki R, Malireddi RKS, Banoth B, Place DE, Briard B, Sharma BR, Tuladhar S, Samir P, Burton A, Kanneganti T-D (2020). Identification of the PANoptosome: a molecular platform triggering pyroptosis, apoptosis, and necroptosis (PANoptosis). Front Cell Infect Microbiol.

[114] Kuriakose T, Man SM, Malireddi RK, Karki R, Kesavardhana S, Place DE (2016). ZBP1/DAI is an innate sensor of influenza virus triggering the NLRP3 inflammasome and programmed cell death pathways. Sci Immunol..

[115] Pandian N, Kanneganti TD (2022). PANoptosis: A unique innate immune inflammatory cell death modality. J Immunol.

[116] Gullett JM, Tweedell RE, Kanneganti TD (2022). It's All in the PAN: Crosstalk, plasticity, redundancies, switches, and interconnectedness encompassed by panoptosis underlying the totality of cell death-associated biological effects. Cells..

[117] Zheng M, Karki R, Vogel P, Kanneganti TD (2020). Caspase-6 is a key regulator of innate immunity, inflammasome activation and host defense. Cell.

[118] Lee S, Karki R, Wang Y, Nguyen LN, Kalathur RC, Kanneganti TD (2021). AIM2 forms a complex with pyrin and ZBP1 to drive PANoptosis and host defence. Nature.

[119] McNab F, Mayer-Barber K, Sher A, Wack A, O'Garra A (2015). Type I interferons in infectious disease. Nat Rev Immunol.

[120] Blanco-Melo D, Nilsson-Payant BE, Liu W-C, Uhl S, Hoagland D, Møller R (2020). Imbalanced host response to SARS-CoV-2 drives development of COVID-19. Cell.

[121] Lucas C, Wong P, Klein J, Castro TBR, Silva J, Sundaram M (2020). Longitudinal analyses reveal immunological misfiring in severe COVID-19. Nature.

[122] Galani IE, Rovina N, Lampropoulou V, Triantafyllia V, Manioudaki M, Pavlos E (2021). Untuned antiviral immunity in COVID-19 revealed by temporal type I/III interferon patterns and flu comparison. Nat Immunol.

[123] Pairo-Castineira E, Clohisey S, Klaric L, Bretherick AD, Rawlik K, Pasko D (2021). Genetic mechanisms of critical illness in COVID-19. Nature.

[124] Zhang Q, Matuozzo D, Le Pen J, Lee D, Moens L, Asano T (2022). Recessive inborn errors of type I IFN immunity in children with COVID-19 pneumonia. J Exp Med.

[125] Bastard P, Rosen LB, Zhang Q, Michailidis E, Hoffmann HH, Zhang Y (2020). Autoantibodies against type I IFNs in patients with life-threatening COVID-19. Science.

[126] Chang SE, Feng A, Meng W, Apostolidis SA, Mack E, Artandi M (2021). New-onset IgG autoantibodies in hospitalized patients with COVID-19. Nat Commun.

[127] Wang EY, Mao T, Klein J, Dai Y, Huck JD, Jaycox JR (2021). Diverse functional autoantibodies in patients with COVID-19. Nature.

[128] Bastard P, Gervais A, Le Voyer T, Rosain J, Philippot Q, Manry J (2021). Autoantibodies neutralizing type I IFNs are present in ~4% of uninfected individuals over 70 years old and account for ~20% of COVID-19 deaths. Sci Immunol..

[129] Zhang Q, Bastard P, Cobat A, Casanova JL (2022). Human genetic and immunological determinants of critical COVID-19 pneumonia. Nature.

[130] Burke JM, St Clair LA, Perera R, Parker R (2021). SARS-CoV-2 infection triggers widespread host mRNA decay leading to an mRNA export block. RNA.

[131] Liu G, Lee JH, Parker ZM, Acharya D, Chiang JJ, van Gent M (2021). ISG15-dependent activation of the sensor MDA5 is antagonized by the SARS-CoV-2 papain-like protease to evade host innate immunity. Nat Microbiol.

[132] Li JY, Liao CH, Wang Q, Tan YJ, Luo R, Qiu Y (2020). The ORF6, ORF8 and nucleocapsid proteins of SARS-CoV-2 inhibit type I interferon signaling pathway. Virus Res.

[133] Chen K, Xiao F, Hu D, Ge W, Tian M, Wang W (2020). SARS-CoV-2 nucleocapsid protein interacts with RIG-I and represses RIG-mediated IFN-β production. Viruses.

[134] Wu J, Shi Y, Pan X, Wu S, Hou R, Zhang Y (2021). SARS-CoV-2 ORF9b inhibits RIG-I-MAVS antiviral signaling by interrupting K63-linked ubiquitination of NEMO. Cell Rep.

[135] Han L, Zhuang MW, Deng J, Zheng Y, Zhang J, Nan ML (2021). SARS-CoV-2 ORF9b antagonizes type I and III interferons by targeting multiple components of the RIG-I/MDA-5-MAVS, TLR3-TRIF, and cGAS-STING signaling pathways. J Med Virol.

[136] Znaidia M, Demeret C, van der Werf S, Komarova AV (2022). Characterization of SARS-CoV-2 evasion: interferon pathway and therapeutic options. Viruses.

[137] Miorin L, Kehrer T, Sanchez-Aparicio MT, Zhang K, Cohen P, Patel RS (2020). SARS-CoV-2 Orf6 hijacks Nup98 to block STAT nuclear import and antagonize interferon signaling. Proc Natl Acad Sci U S A.

[138] Sui L, Zhao Y, Wang W, Wu P, Wang Z, Yu Y (2021). SARS-CoV-2 membrane protein inhibits type I interferon production through ubiquitin-mediated degradation of TBK1. Front Immunol.

[139] Thoms M, Buschauer R, Ameismeier M, Koepke L, Denk T, Hirschenberger M (2020). Structural basis for translational shutdown and immune evasion by the Nsp1 protein of SARS-CoV-2. Science.

[140] Hsu JC, Laurent-Rolle M, Pawlak JB, Wilen CB, Cresswell P (2021). Translational shutdown and evasion of the innate immune response by SARS-CoV-2 NSP14 protein. Proc Natl Acad Sci U S A.

[141] Mao T, Israelow B, Lucas C, Vogels CBF, Gomez-Calvo ML, Fedorova O (2022). A stem-loop RNA RIG-I agonist protects against acute and chronic SARS-CoV-2 infection in mice. J Exp Med.

[142] Bessière P, Wasniewski M, Picard-Meyer E, Servat A, Figueroa T, Foret-Lucas C (2021). Intranasal type I interferon treatment is beneficial only when administered before clinical signs onset in the SARS-CoV-2 hamster model. PLoS Pathog.

[143] Kalil AC, Mehta AK, Patterson TF, Erdmann N, Gomez CA, Jain MK (2021). Efficacy of interferon beta-1a plus remdesivir compared with remdesivir alone in hospitalised adults with COVID-19: a double-bind, randomised, placebo-controlled, phase 3 trial. Lancet Respir Med.

[144] Wang N, Zhan Y, Zhu L, Hou Z, Liu F, Song P (2020). Retrospective multicenter cohort study shows early interferon therapy is associated with favorable clinical responses in COVID-19 patients. Cell Host Microbe.

[145] Dinnon KH, Leist SR, Schäfer A, Edwards CE, Martinez DR, Montgomery SA (2020). A mouse-adapted model of SARS-CoV-2 to test COVID-19 countermeasures. Nature.

[146] Jagannathan P, Andrews JR, Bonilla H, Hedlin H, Jacobson KB, Balasubramanian V (2021). Peginterferon Lambda-1a for treatment of outpatients with uncomplicated COVID-19: a randomized placebo-controlled trial. Nat Commun.

[147] WHO Solidarity Trial Consortium (2022). Remdesivir and three other drugs for hospitalised patients with COVID-19: final results of the WHO solidarity randomised trial and updated meta-analyses. Lancet.

[148] Major J, Crotta S, Llorian M, McCabe TM, Gad HH, Priestnall SL (2020). Type I and III interferons disrupt lung epithelial repair during recovery from viral infection. Science.

[149] Banoth B, Tuladhar S, Karki R, Sharma BR, Briard B, Kesavardhana S (2020). ZBP1 promotes fungi-induced inflammasome activation and pyroptosis, apoptosis, and necroptosis (PANoptosis). J Biol Chem.

[150] Hadjadj J, Yatim N, Barnabei L, Corneau A, Boussier J, Smith N (2020). Impaired type I interferon activity and inflammatory responses in severe COVID-19 patients. Science.

[151] Doherty GM, Lange JR, Langstein HN, Alexander HR, Buresh CM, Norton JA (1992). Evidence for IFN-gamma as a mediator of the lethality of endotoxin and tumor necrosis factor-alpha. J Immunol.

[152] Ackermann M, Verleden SE, Kuehnel M, Haverich A, Welte T, Laenger F (2020). Pulmonary vascular endothelialitis, thrombosis, and angiogenesis in Covid-19. N Engl J Med.

[153] Diorio C, Henrickson SE, Vella LA, McNerney KO, Chase J, Burudpakdee C (2020). Multisystem inflammatory syndrome in children and COVID-19 are distinct presentations of SARS–CoV-2. J Clin Invest.

[154] Belhadjer Z, Méot M, Bajolle F, Khraiche D, Legendre A, Abakka S (2020). Acute heart failure in multisystem inflammatory syndrome in children (MIS-C) in the context of global SARS-CoV-2 pandemic. Circulation.

[155] Esmon CT (2005). The interactions between inflammation and coagulation. Br J Haematol.

[156] Bhattacharjee S, Banerjee M (2020). Immune thrombocytopenia secondary to COVID-19: a systematic review. SN Compr Clin Med.

[157] Kaneko N, Kuo H-H, Boucau J, Farmer JR, Allard-Chamard H, Mahajan VS (2020). Loss of Bcl-6-expressing T follicular helper cells and germinal centers in COVID-19. Cell.

[158] Ohta A, Sekimoto M, Sato M, Koda T, Nishimura SI, Iwakura Y (2000). Indispensable role for TNF- and IFN- at the effector phase of liver injury mediated by Th1 cells specific to hepatitis B virus surface antigen. J Immunol.

[159] Zhu L, Yang P, Zhao Y, Zhuang Z, Wang Z, Song R (2020). Single-cell sequencing of peripheral mononuclear cells reveals distinct immune response landscapes of COVID-19 and influenza patients. Immunity.

[160] Simpson DS, Pang J, Weir A, Kong IY, Fritsch M, Rashidi M (2022). Interferon-γ primes macrophages for pathogen ligand-induced killing via a caspase-8 and mitochondrial cell death pathway. Immunity.

[161] Zhang F, Mears JR, Shakib L, Beynor JI, Shanaj S, Korsunsky I (2021). IFN-γ and TNF-α drive a CXCL10+ CCL2+ macrophage phenotype expanded in severe COVID-19 lungs and inflammatory diseases with tissue inflammation. Genome Med.

[162] Xu Z, Shi L, Wang Y, Zhang J, Huang L, Zhang C (2020). Pathological findings of COVID-19 associated with acute respiratory distress syndrome. Lancet Respir Med.

[163] Schulte-Schrepping J, Reusch N, Paclik D, Baßler K, Schlickeiser S, Zhang B (2020). Severe COVID-19 is marked by a dysregulated myeloid cell compartment. Cell.

[164] Silvin A, Chapuis N, Dunsmore G, Goubet AG, Dubuisson A, Derosa L (2020). Elevated calprotectin and abnormal myeloid cell subsets discriminate severe from mild COVID-19. Cell.

[165] Perico L, Benigni A, Casiraghi F, Ng LFP, Renia L, Remuzzi G (2021). Immunity, endothelial injury and complement-induced coagulopathy in COVID-19. Nat Rev Nephrol.

[166] Liao M, Liu Y, Yuan J, Wen Y, Xu G, Zhao J (2020). Single-cell landscape of bronchoalveolar immune cells in patients with COVID-19. Nat Med.

[167] Delorey TM, Ziegler CGK, Heimberg G, Normand R, Yang Y, Segerstolpe Å (2021). COVID-19 tissue atlases reveal SARS-CoV-2 pathology and cellular targets. Nature.

[168] Xydakis MS, Albers MW, Holbrook EH, Lyon DM, Shih RY, Frasnelli JA (2021). Post-viral effects of COVID-19 in the olfactory system and their implications. Lancet Neurol.

[169] Livanos AE, Jha D, Cossarini F, Gonzalez-Reiche AS, Tokuyama M, Aydillo T (2021). Intestinal host response to SARS-CoV-2 infection and COVID-19 outcomes in patients with gastrointestinal symptoms. Gastroenterology.

[170] Merad M, Blish CA, Sallusto F, Iwasaki A (2022). The immunology and immunopathology of COVID-19. Science.

[171] Forman R, Shah S, Jeurissen P, Jit M, Mossialos E (2021). COVID-19 vaccine challenges: What have we learned so far and what remains to be done?. Health Policy.

[172] U.S. Food and Drug Administration. Emergency Use Authorization. 2022.

[173] Horby P, Lim WS, Emberson JR, Mafham M, Bell JL, Linsell L (2021). Dexamethasone in hospitalized patients with Covid-19. N Engl J Med.

[174] Salama C, Han J, Yau L, Reiss WG, Kramer B, Neidhart JD (2021). Tocilizumab in patients hospitalized with Covid-19 pneumonia. N Engl J Med.

[175] Lescure FX, Honda H, Fowler RA, Lazar JS, Shi G, Wung P (2021). Sarilumab in patients admitted to hospital with severe or critical COVID-19: a randomised, double-blind, placebo-controlled, phase 3 trial. Lancet Respir Med.

[176] Rosas IO, Diaz G, Gottlieb RL, Lobo SM, Robinson P, Hunter BD (2021). Tocilizumab and remdesivir in hospitalized patients with severe COVID-19 pneumonia: a randomized clinical trial. Intensive Care Med.

[177] Rosas IO, Bräu N, Waters M, Go RC, Hunter BD, Bhagani S (2021). Tocilizumab in hospitalized patients with severe Covid-19 pneumonia. N Engl J Med.

[178] Sancho-López A, Caballero-Bermejo AF, Ruiz-Antorán B, Múñez Rubio E, García Gasalla M, Buades J (2021). Efficacy and safety of sarilumab in patients with COVID19 pneumonia: a randomized, phase III clinical trial (SARTRE Study). Infect Dis Ther.

[179] Laing AG, Lorenc A, Del Molino Del Barrio I, Das A, Fish M, Monin L (2020). A dynamic COVID-19 immune signature includes associations with poor prognosis. Nat Med.

[180] Cavalli G, De Luca G, Campochiaro C, Della-Torre E, Ripa M, Canetti D (2020). Interleukin-1 blockade with high-dose anakinra in patients with COVID-19, acute respiratory distress syndrome, and hyperinflammation: a retrospective cohort study. Lancet Rheumatol.

[181] Generali D, Bosio G, Malberti F, Cuzzoli A, Testa S, Romanini L (2021). Canakinumab as treatment for COVID-19-related pneumonia: a prospective case-control study. Int J Infect Dis.

[182] Caricchio R, Abbate A, Gordeev I, Meng J, Hsue PY, Neogi T (2021). Effect of canakinumab vs placebo on survival without invasive mechanical ventilation in patients hospitalized with severe COVID-19: a randomized clinical trial. JAMA.

[183] Kyriazopoulou E, Panagopoulos P, Metallidis S, Dalekos GN, Poulakou G, Gatselis N (2021). An open label trial of anakinra to prevent respiratory failure in COVID-19. Elife.

[184] Pontali E, Volpi S, Signori A, Antonucci G, Castellaneta M, Buzzi D (2021). Efficacy of early anti-inflammatory treatment with high doses of intravenous anakinra with or without glucocorticoids in patients with severe COVID-19 pneumonia. J Allergy Clin Immunol.

[185] Kyriazopoulou E (2021). Early treatment of COVID-19 with anakinra guided by soluble urokinase plasminogen receptor plasma levels: a double-blind, randomized controlled phase 3 trial. Nat Med..

[186] Lopes MI, Bonjorno LP, Giannini MC, Amaral NB, Menezes PI, Dib SM (2021). Beneficial effects of colchicine for moderate to severe COVID-19: a randomised, double-blinded, placebo-controlled clinical trial. RMD Open.

[187] National Institues of Health. COVID-19 treatment guidelines: colchicine. 2022.

[188] Gianfrancesco M, Hyrich KL, Al-Adely S, Carmona L, Danila MI, Gossec L (2020). Characteristics associated with hospitalisation for COVID-19 in people with rheumatic disease: data from the COVID-19 Global Rheumatology Alliance physician-reported registry. Ann Rheum Dis.

[189] Kalil AC, Patterson TF, Mehta AK, Tomashek KM, Wolfe CR, Ghazaryan V (2021). Baricitinib plus remdesivir for hospitalized adults with COVID-19. N Engl J Med.

[190] Phetsouphanh C, Darley DR, Wilson DB, Howe A, Munier CML, Patel SK (2022). Immunological dysfunction persists for 8 months following initial mild-to-moderate SARS-CoV-2 infection. Nat Immunol.

[191] Ayoubkhani D, Bermingham C, Pouwels KB, Glickman M, Nafilyan V, Zaccardi F (2022). Trajectory of long covid symptoms after COVID-19 vaccination: community based cohort study. BMJ.

